# Relationship Between Sleep–Wake Disturbance and Risk of Malnutrition in Hospitalized Patients With Cirrhosis

**DOI:** 10.3389/fnut.2021.719176

**Published:** 2021-08-31

**Authors:** Yangyang Hui, Xiaoyu Wang, Zihan Yu, Hongjuan Feng, Chaoqun Li, Lihong Mao, Xiaofei Fan, Lin Lin, Binxin Cui, Xin Chen, Longhao Sun, Bangmao Wang, Chao Sun

**Affiliations:** ^1^Department of Gastroenterology and Hepatology, Tianjin Medical University General Hospital, Tianjin, China; ^2^Tianjin Medical University General Hospital, Tianjin Institute of Digestive Disease, Tianjin, China; ^3^Department of Nutriology, Tianjin Third Central Hospital, Tianjin, China; ^4^Department of Internal Medicine, Tianjin Hexi Hospital, Tianjin, China; ^5^Department of Gastroenterology, Tianjin Medical University General Hospital Airport Hospital, Tianjin, China; ^6^Department of General Surgery, Tianjin Medical University General Hospital, Tianjin, China

**Keywords:** malnutrition, PSQI, RFH-NPT, sleep-wake disturbance, liver cirrhosis

## Abstract

Both sleep–wake disturbance and malnutrition are common in cirrhosis and might be associated with similar adverse outcomes, such as impaired health-related quality of life, hepatic encephalopathy, and sarcopenia, but there is no study investigating the relationship between these two. We aimed to explore the relationship between sleep–wake disturbance [estimated by the Pittsburgh Sleep Quality Index (PSQI)] and malnutrition risk [estimated by the Royal Free Hospital-Nutritional Prioritizing Tool (RFH-NPT)]. About 150 patients with cirrhosis were prospectively recruited. The nutritional risk is classified as low (0 points), moderate (1 point), and high (2–7 points) according to the RFH-NPT score. A global PSQI >5 indicated poor sleepers. Furthermore, multivariate linear regression analyses were performed to determine the relationship between *sleep–wake* disturbance and malnutrition. The median PSQI was seven, and RFH-NPT was two in the entire cohort, with 60.67 and 56.67% rated as poor sleep quality and high malnutrition risk, respectively. Patients with cirrhosis with poor sleep quality had significantly higher RFH-NPT score (3 vs. 1, *P* = 0.007). Our multivariate analyses indicated that male patients (β = 0.279, *P* < 0.001), ascites (β = 0.210, *P* = 0.016), and PSQI (β = 0.262, *P* = 0.001) were independent predictors of malnutrition. In addition, the differences regarding PSQI score were more significant in male patients, as well as those >65 years or with Child-Turcotte-Pugh class A/B (CTP-A/B) or the median model for end-stage liver disease (MELD) <15. Taken together, the sleep–wake disturbance is strongly correlated with high malnutrition risk in patients with cirrhosis. Given sleep–wake disturbance is remediable, it is tempting to incorporate therapies to reverse poor sleep quality for improving nutritional status in patients with cirrhosis.

## Introduction

Sleep–wake disturbance is a common feature of cirrhosis and advanced chronic liver diseases. It has been estimated that approximately 60% or more of patients with cirrhosis regarded themselves as poor sleepers, which was determined by the Pittsburgh Sleep Quality Index (PSQI) (1). The causality between deteriorating sleep and other predisposing factors in patients with cirrhosis is still under extensive investigation with inconsistent data. Montagnese et al. showed sleep deterioration assessed by PSQI is irrelevant to the presence/degree of hepatic encephalopathy (HE) (2). Conversely, another study implicated that sleep quality, with 24-h polysomnography (PSG), improves in parallel with the amelioration of HE (3). Furthermore, sleep–wake disturbance could negatively and independently affect health-related quality of life (HRQoL) and increase the risk of developing hepatic malignancies (4, 5).

Malnutrition is another cirrhosis-associated complication, which could result in an increased risk of liver failure, infection, higher prevalence of complications due to portal hypertension, and prolonged hospitalized stays (6). The prevalence of malnutrition dramatically increased in correspondence with aggravated liver function, while more than half the patients with cirrhosis represent malnourished and concomitant decompensated insults (7). More recently, several studies have investigated the relationships between sleep disorders and malnutrition risk in distinct pathological entities. Notably, Soysal et al. implicated a close association between moderate/severe insomnia and the presence of malnutrition as well as high malnutrition risk in elders (8). Another study found that sleep disorders are significantly correlated with malnutrition risk in older adults (9). Given these two complications might share multiple converging mechanisms and lead to similar outcomes, we speculate that poor sleep quality might be associated with a high risk of malnutrition in cirrhotics.

As far as we can determine, there is a paucity of data exploring the association between sleep–wake disturbance and nutritional state among hospitalized cirrhotics. Collectively, the present study aimed to (1) analyze the association between sleep quality and nutritional state; and (2) clarify the differences regarding PSQI scores in terms of malnutrition risk across various subgroups.

## Materials and Methods

### Study Cohort

Patients with cirrhosis aged ≥18 years, who were hospitalized between December 2019 and January 2021, were prospectively enrolled for the current study. Those with concomitant malignancies, severe HE (*via* the time to finish a numbers connection test of >120 s), and presence with acute-on-chronic liver failure were excluded (10, 11). In concert with previous work conducted by Ghabril et al., we did not exclude patients with active alcohol since they contribute to a significant subset of patients with cirrhosis (4). In our center, HE was detected from the time to complete the number connection test performed upon hospitalization and categorized as present if ≥60 s needed to complete the test (10, 12, 13). The diagnosis of liver cirrhosis was based on medical history, laboratory examinations, imaging results, endoscopic data, and/or liver biopsy.

### Sleep Quality

We evaluated the sleep quality using a Chinese version of the PSQI for screening sleep–wake disturbance (14). The reliability and validity of PSQI have gained broad acceptance worldwide. It comprises a total of 10 questions ranging from seven components of sleep patterns, namely, subjective sleep quality, sleep latency, sleep duration, habitual sleep efficiency, sleep disturbances, sleep medication use, and daytime dysfunction. Each component is scored on 0–3 points. The sum of the scores of all seven categories composes the total PSQI score. A higher score unravels poorer sleep quality, where a global PSQI score >5 has been verified to discriminate between poor from good sleeper (15).

### Royal Free Hospital-Nutritional Prioritizing Tool

The RFH-NPT scores were calculated according to our previous depiction (16). Briefly, the risk of malnutrition was categorized into low (0 points), moderate (1 point), and high (2–7 points) in terms of RFH-NPT scores. Initially, we inquired and recorded the presence of tube feeding or acute alcoholic hepatitis, because these medical issues might predispose subjects to highly malnourished conditions. Next, we clarified between the groups of patients present with or without edema/ascites. At last, the total scores were summarized, and individuals were demarcated to the corresponding risk groups.

### Clinical and Laboratory Metrics

Details in relation to clinical and laboratory results have been explicitly introduced in our previous publication (17). Because a large number of patients with cirrhosis presented with fluid retention, it is more reasonable to calculate dry weight for assessing body mass index (BMI). We calculated the dry weight by subtracting 5% for mild ascites, 10% for moderate ascites, and 15% for bulky ascites for subjects with edema and ascites, and 5% of body weight was subtracted for patients with peripheral edema (18).

### Statistical Analyses

Descriptive statistics were presented as mean ± SD, median [interquartile range (IQR)], proportions, or simple frequencies as appropriate. Continuous data were compared by an independent Student's *t*-test or Mann–Whitney *U* test in cases without normal distribution. Multiple comparisons were performed by using the one-way ANOVA or the Kruskal–Wallis test with Dunn's post-*hoc* test. The univariate analysis accounted for the correlation that exists between demographic/laboratory parameters, PSQI scores, and RFH-NPT scores. Multivariate linear regression analysis was implemented to figure out the independent factors associated with the risk of malnutrition as measured by RFH-NPT. We regarded *P* < 0.05 as statistically significant. All statistical analyses were carried out by using SPSS 21.0 (IBM, New York, NY, USA) and Graphpad Prism 8.0.1 (La Jolla, CA, USA).

## Results

Table 1 describes the baseline characteristics of the study population separated by PSQI. A total of 150 patients with cirrhosis (male patients: *n* = 70, 46.67%) with a mean age of 61.24 ± 10.31 years were recruited to the investigation. The etiologies of cirrhosis were due to chronic viral infection in 38 (25.33%), alcohol in 35 (23.33%), autoimmune/cholestatic liver disease in 46 (30.67%), and cryptogenic/non-alcoholic fatty liver disease (NAFLD) in 31 participants (20.67%). Ninety-three patients (62.00%) presented with ascites upon admission, while this number was 13 with HE (8.67%). Among the overall subjects, 48 (32.00%) were classified as Child-Turcotte-Pugh (CTP) class A, 82 (54.67%) as CTP-B, and 20 as CTP-C (13.33%). The median model for end-stage liver disease (MELD) score was 9.6 (IQR, 7.2–12.2). When stratified by PSQI as good and poor sleepers, a total of 91 cirrhotics (60.67%) were classified as poor sleepers with PSQI of <5 points. Intriguingly, our results indicated that the poor sleepers have lower BMI (22.89 vs. 24.69 kg/m^2^, *P* = 0.003), more ascites (69.23 vs. 50.85%, *P* = 0.024), and higher RFH-NPT scores (3 vs. 1 points, *P* = 0.007).

**Table 1:** Baseline characteristics of cirrhotic patients classified by PSQI score.

	**Total (*n* = 150)**	**Good sleepers (*n* = 59)**	**Poor sleepers (*n* = 91)**	***P***
Age (years)	61.24 ± 10.31	59.83 ± 12.43	62.19 ± 8.56	0.628
Gender, *n* (%)				0.246
Male	70 (46.67)	31 (52.54)	39 (42.86)	
Female	80 (53.33)	28 (47.46)	52 (57.14)	
BMI (kg/m^2^)	23.44 (20.29, 26.17)	24.69 (22.19, 27.43)	22.89 (19.37, 25.39)	0.003
Etiology, *n* (%)				0.945
Viral infection	38 (25.33)	18 (30.51)	20 (21.98)	
Alcohol	35 (23.33)	11 (18.64)	24 (26.37)	
AILD/Cholestatic	46 (30.67)	17 (28.81)	29 (31.87)	
Cryptogenic/NAFLD	31 (20.67)	13 (22.04)	18 (19.78)	
Ascites, *n* (%)	93 (62.00)	30 (50.85)	63 (69.23)	0.024
Hepatic encephalopathy, *n* (%)	13 (8.67)	6 (10.17)	7 (7.69)	0.768
CTP score	7 (6,9)	7 (6,9)	7 (6,9)	0.339
CTP class, *n* (%)				0.336
A	48 (32.00)	23 (38.98)	25 (27.47)	
B	82 (54.67)	29 (49.15)	53 (58.24)	
C	20 (13.33)	7 (11.86)	13 (14.29)	
RFH-NPT	2 (0, 6)	1 (0, 4)	3 (1,6)	0.007
MELD	9.6 (7.2, 12.2)	9.9 (7.8, 11.9)	9.5 (5.8, 12.6)	0.610
Na (mmol/L)	140 (137, 142)	140 (138, 142)	140 (137, 142)	0.621
K (mmol/L)	3.9 (3.5, 4.1)	3.8 (3.5, 4.1)	3.9 (3.5, 4.1)	0.867
Albumin (g/L)	28 (25,32)	28 (26,33)	28 (24,32)	0.311
PT-INR	1.28 (1.18, 1.44)	1.3 (1.19, 1.48)	1.26 (1.17, 1.42)	0.238
Hemoglobin (g/L)	86 (70, 109)	88 (73, 114.00)	85 (66, 109)	0.291
ALT (U/L)	22 (15,36)	23 (15,33)	21 (15,37)	0.703
Total bilirubin (μmol/L)	21.60 (13.20, 37.50)	19.35 (11.85,42.13)	21.00 (14.00, 37.40)	0.479

*Data were expressed as mean ± standard deviation, median (interquartile range), proportions or simple frequencies as appropriate*.

*We classify all cirrhotic patients into good sleepers (PSQI ≤ 5) and poor sleepers (PSQI > 5)*.

*PSQI, Pittsburgh Sleep Quality Index; BMI, body mass index; AILD, autoimmune liver disease; NAFLD, non-alcoholic fatty liver disease; CTP, Child-Turcotte-Pugh; RFH-NPT, Royal Free Hospital-Nutritional Prioritizing Tool; MELD, model for end-stage liver disease; PT-INR, prothrombin-international normalized ratio; ALT, alanine aminotransferase*.

Table 2 shows the results of linear regression analyses of the sleep–wake disturbance associated with malnutrition risk estimated by RFH-NPT. Our univariable analyses showed age (β coefficient = 0.187, *P* = 0.024), male (β coefficient = 0.280, *P* = 0.001), ascites (β coefficient = 0.356, *P* < 0.001), PSQI (β coefficient = 0.350, *P* < 0.001), CTP score (β coefficient = 0.268, *P* = 0.001), albumin (β coefficient = −0.165, *P* = 0.049), and sodium (β coefficient = −0.186, *P* = 0.027) were factors associated with original RFH-NPT score with a *P* < 0.05. Further multivariate linear regression implicated that male (β coefficient = 0.279, *P* < 0.001), ascites (β coefficient = 0.210, *P* = 0.016), and PSQI (β coefficient = 0.262, *P* = 0.001) were independent factors for malnutrition risk as indicated by RFH-NPT.

**Table 2:** A multivariate linear regression analysis to assess association between covariates and RFH-NPT.

**Coefficients for the associations with RFH-NPT**						
	**Simple regression**			**Multiple regression**		
**Variable**	**β**	**95% CI**	***P***	**β**	**95% CI**	***P***
Age (years)	0.187	0.025, 0.348	0.024	0.115	−0.034, 0.264	0.130
Gender (Male)	0.280	−0.437, −0.123	0.001	0.279	0.423,−0.134	<0.001
BMI (kg/m^2^)	−0.122	−0.289, 0.043	0.146			
Ascites	0.356	0.203, 0.508	<0.001	0.210	0.039, 0.377	0.016
PSQI	0.350	0.196, 0.502	<0.001	0.262	0.114, 0.404	0.001
CTP score	0.268	0.108, 0.431	0.001	0.099	−0.082, 0.280	0.281
Hemoglobin (g/L)	0.052	−0.115, 0.219	0.538			
Hepatic encephalopathy	−0.032	−0.195, 0.130	0.695			
MELD score	−0.051	−0.217, 0.116	0.550			
Albumin (g/L)	−0.165	−0.329, 0.000	0.049	−0.009	−0.174, 0.156	0.916
ALT(U/L)	−0.092	−0.257, 0.075	0.278			
Na (mmol/L)	−0.186	−0.349, −0.022	0.027	−0.096	−0.246,−0.054	0.210

*RFH-NPT, Royal Free Hospital-Nutritional Prioritizing Tool; BMI, body mass index; PSQI, Pittsburgh Sleep Quality Index; CTP, Child-Turcotte-Pugh; MELD, model for end-stage liver disease; ALT, alanine aminotransferase; CI, confidence interval*.

**Figure 1:**
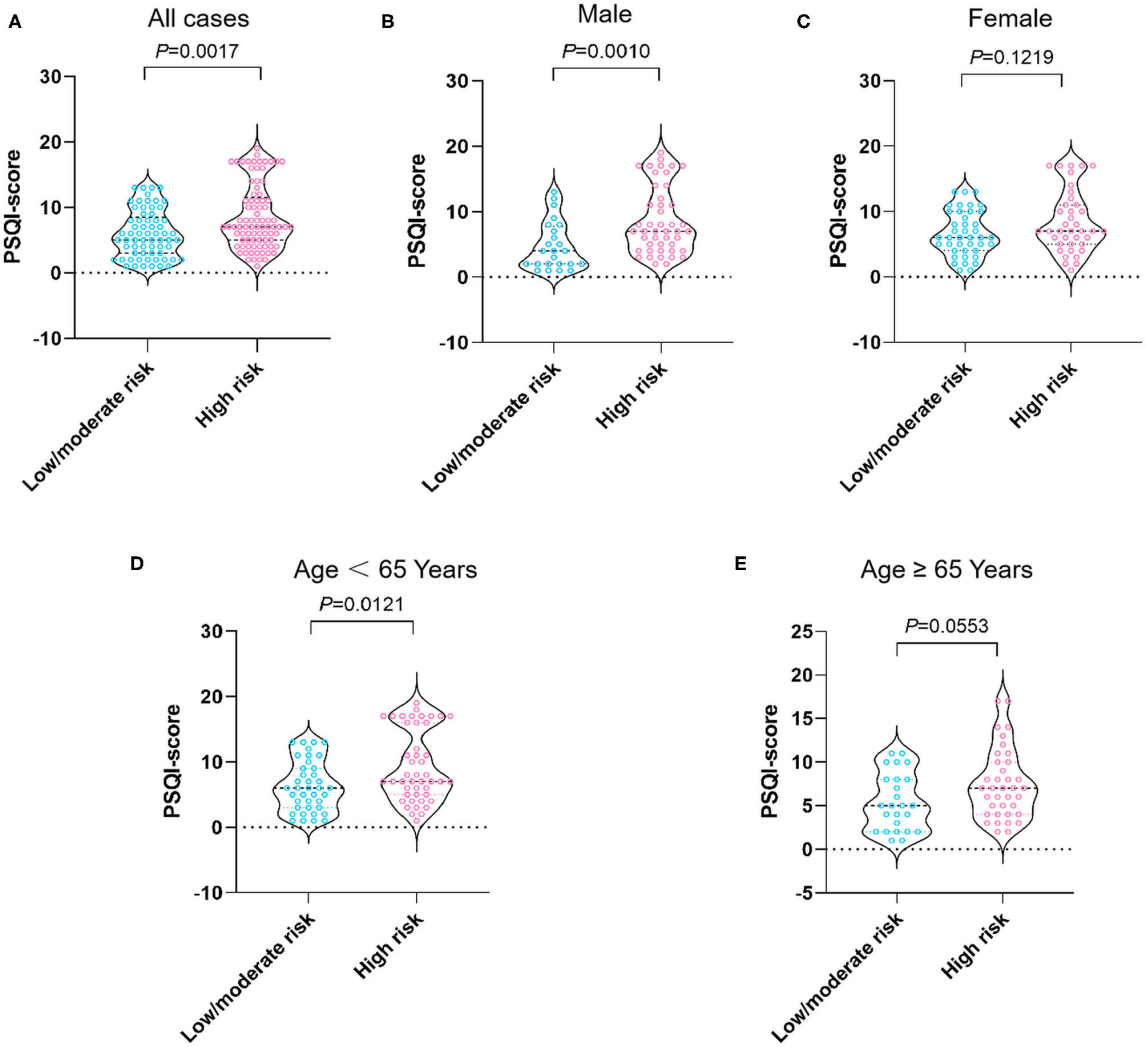
PSQI score stratified by nutritional status in all cases **(A)**, male patients **(B)**, female patients **(C)**, patients aged <65 years **(D)**, and aged 65 years and over **(E)**. The risk of malnutrition was categorized into low (0 points), moderate (1 point), and high (2–7 points) in terms of RFH-NPT scores. PSQI, Pittsburgh Sleep Quality Index; RFH-NPT, Royal Free Hospital-Nutritional Prioritizing Tool.

The median PSQI score in the study population was seven (IQR, 4–10). As shown in Table 3, the patients at high malnutrition risk exhibited the highest PSQI score in comparison with other groups (7 vs. 6 vs. 5, *P* = 0.008). As shown in Figure 1A, the median PSQI score in the high malnutrition risk group was significantly higher than that in the low/moderate malnutrition risk groups (7 vs. 5, *P* = 0.0017). Additionally, the median PSQI score in the high malnutrition risk group was significantly higher than that in the low/moderate malnutrition risk group in male cirrhotics (7 vs. 4, *P* = 0.0010; Figure 1B). Conversely, no significant difference was observed regarding PSQI score between these two groups in female patients (7 vs. 6, *P* = 0.1219; Figure 1C).

**Table 3:** Characteristics of cirrhotic patients classified by RFH-NPT scores.

	**Low risk (*n* = 46)**	**Moderate risk (*n* = 19)**	**High risk (*n* = 85)**	***P***
Age (years)	59.62 ± 11.10	58.21 ± 11.56	62.99 ± 9.33	0.157
Gender, *n* (%)				0.062
Male	15 (32.61)	9 (47.37)	46 (54.12)	
Female	31 (67.39)	10 (52.63)	39 (45.88)	
BMI (kg/m^2^)	25.13 ± 4.31	23.70 ± 4.67	22.85 ± 4.31	0.023
Etiology, *n* (%)				<0.001
Viral infection	15 (32.61)	9 (47.37)	14 (16.47)	
Alcohol	2 (4.35)	2 (10.53)	31 (36.47)	
AILD/cholestatic	15 (32.61)	7 (36.84)	24 (28.24)	
Cryptogenic/NAFLD	14(30.43)	1 (5.26)	16 (18.82)	
Ascites, *n* (%)	15 (32.61)	13 (68.42)	65 (76.47)	<0.001
Hepatic encephalopathy, *n* (%)	4 (8.70)	2 (10.53)	7 (8.24)	0.950
CTP score	7 (6, 8)	7 (6, 9)	8 (7, 9)	0.006
CTP class, *n* (%)				
A	22 (47.83)	7 (36.84)	19 (22.35)	0.039
B	21 (45.65)	9 (47.37)	52 (61.18)	
C	3 (6.52)	3 (15.79)	14 (16.47)	
PSQI	5 (3, 8)	6 (3, 10)	7 (5, 11)	0.008
MELD	9.9 (8.4, 12.4)	10.1 (8.2, 11.3)	9.2 (5.2, 12.3)	0.536
Na (mmol/L)	141 (139, 142)	140 (137, 141)	139 (137, 142)	0.058
Albumin (g/L)	29 (26, 32)	28 (24, 31)	28 (24, 32)	0.255
ALT (U/L)	21 (13, 30)	22 (16, 41)	23 (15, 37)	0.693
Total bilirubin (μmol/L)	16.85 (12.05, 31.03)	22.70 (11.40, 36.90)	22.60 (14.10, 49.03)	0.103
PT-INR	1.23 (1.18, 1.38)	1.26 (1.17, 1.37)	1.3 (1.18, 1.49)	0.566

*Data were expressed as mean ± standard deviation, median (interquartile range), proportions or simple frequencies as appropriate*.

*The risk of malnutrition was categorized into low (0 points), moderate (1 point) or high (2–7 points) in terms of RFH-NPT scores*.

*RFH-NPT, Royal Free Hospital-Nutritional Prioritizing Tool; BMI, body mass index; AILD, autoimmune liver disease; NAFLD, non-alcoholic fatty liver disease; CTP, Child-Turcotte-Pugh; PSQI, Pittsburgh Sleep Quality Index; MELD, model for end-stage liver disease; PT-INR, prothrombin-international normalized ratio; ALT, alanine aminotransferase*.

**Figure 2:**
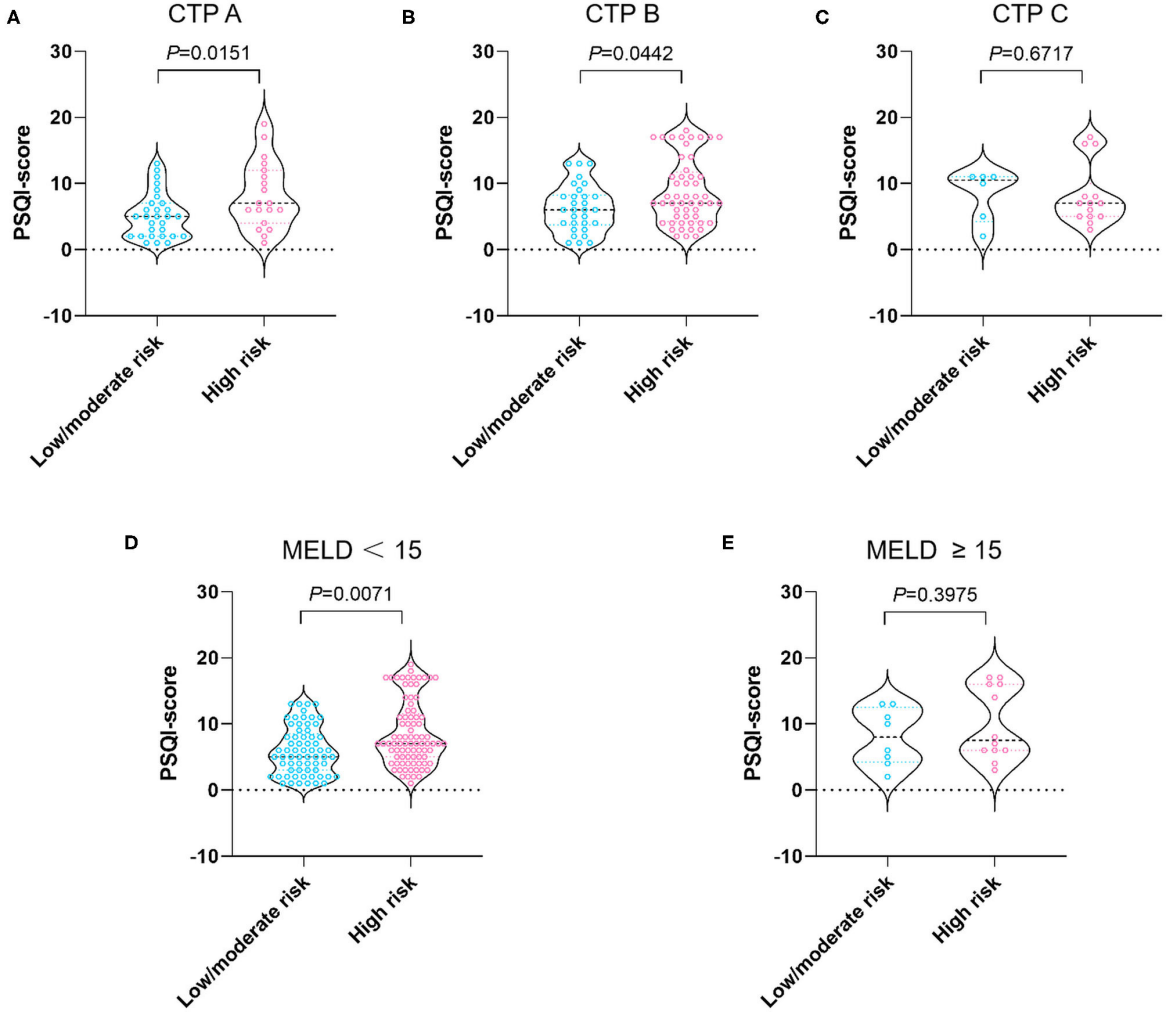
PSQI score stratified by nutritional status in patients with CTP-A **(A)**, CTP-B **(B)**, CTP-C **(C)**, MELD <15 **(D)**, and MELD ≥ 15 points **(E)**. The risk of malnutrition was categorized into low (0 points), moderate (1 point), and high (2–7 points) in terms of RFH-NPT scores. PSQI, Pittsburgh Sleep Quality Index; RFH-NPT, Royal Free Hospital-Nutritional Prioritizing Tool; CTP, Child-Turcotte-Pugh; MELD, model for end-stage liver disease.

As shown in Figures 1D,E, when stratified by age we showed that the median PSQI score in the high malnutrition risk group was markedly higher than that in the low/moderate malnutrition risk group in patients with cirrhosis <65 years (7 vs. 6, *P* = 0.0121). On the other hand, the median PSQI score in the high malnutrition risk group had a tendency toward significance in comparison with that in the low/moderate malnutrition risk group in patients with cirrhosis aged ≥65 years (7 vs. 5, *P* = 0.0553). Notably, the median PSQI scores in the high malnutrition risk group were significantly higher than those in the low/moderate malnutrition risk group rated as CTP-A (7 vs. 5, *P* = 0.0151), CTP-B (7 vs. 6, *P* = 0.0442), and with MELD <15 points (7 vs. 5, *P* = 0.0071; Figure 2).

## Discussion

Our present study explored the relationship between sleep–wake disturbance and malnutrition risk in hospitalized patients with cirrhosis, and the RFH-NPT score was observed to be significantly higher in poor sleepers. Moreover, PSQI has been demonstrated to be an independent risk factor positively correlated with RFH-NPT, namely, a high risk of malnutrition. In addition, the differences with respect to PSQI scores were markedly pronounced in male patients as well as those who were <65 years or with less deteriorating liver function.

Mounting evidence has addressed that both sleep–wake disturbance and malnutrition appear to be prevalent in patients with cirrhosis (19, 20). From the clinical perspective, sleep–wake disturbance might negatively impact HRQoL, depression, and psychological distress (4, 21). Notably, PSQI increased in parallel with HE, and patients with cirrhosis with higher PSQI scores suffered from worse HRQoL (22). More recently, a systemic review of 109 studies intended to comprehensively summarize the factors relevant to poor HRQoL in patients with cirrhosis (23). Their findings showed that malnutrition, as a modifiable issue, is among the top factors, which are associated with impairment in HRQoL in most studies. Collectively, since sleep–wake disturbance and malnutrition can lead to similarly adverse outcomes, and both are prevalent in cirrhotics; it is imperative to explore the interaction between these two complications.

In fact, some pioneering scholars in the field of geriatrics have already corroborated a close relationship between various sleep–wake abnormalities and malnutrition in older adults. Tuna et al. reported a negative correlation between the Simplified Nutritional Assessment Questionnaire score (perception of appetite, taste of food, portion of a meal enough for subjects to feel full, and number of daily meals) and the global PSQI score, which unravels the elderly with poor sleep quality exhibit a higher risk of weight loss (24). Another study showed that insomnia is significantly correlated with malnourished status and associated with a low Mini Nutritional Assessment score (8). A study recruiting 6,792 community-dwelling older adults in West China indicated that poor sleepers determined by PSQI >5 are associated with 162% higher risk of malnutrition (odds ratio [OR]: 1.62, 95% CI, 1.44–1.82) compared with good sleepers (9). However, there are scant data regarding the relationship between sleep disturbance and malnutrition risk in cirrhotics.

An important finding of the current investigation was that sleep–wake disturbance represents an independent risk factor for RFH-NPT, which refers to the risk of malnutrition, after adjusting for confounding variables. The standardized coefficient is noted with the strongest value (β = 0.262, *P* = 0.001) in comparison with other modifiable covariates (ascites: β = 0.210, *P* = 0.016). Therefore, it is tempting to effectively reverse disturbed sleep to improve malnourishment. As a matter of fact, a randomized, placebo-controlled trial conducted by Sharma et al. clearly showed that 5 mg/day zolpidem for 4 weeks in CTP-A/B patients with cirrhosis and insomnia results in significant increases in total sleep time, sleep efficiency, and improvement in polysomnographic parameters of sleep initiation and maintenance (25).

How might impaired sleep quality lead to high malnutrition risk? We offer several possible mechanisms for this pathway. First, chronic inflammation has been widely proved to be related to sleep–wake disturbance and elevated inflammatory cytokines might regulate and modulate sleep–waking behavior among patients with cirrhosis (26). For instance, Tsai et al. found serum interleukin-6 (IL-6) and tumor necrosis factor-alpha (TNF-α) levels are remarkably elevated in poor sleepers (PSQI > 5), and IL-6 appears to be an independent predictor of poor sleep quality (27). On the other hand, extensive and persistent inflammatory milieu also predisposes individuals to malnourished conditions *via* increased muscle catabolism and resting energy expenditure (28). In a word, inflammation might serve as an upstream factor influencing both sleep–wake disturbance and malnutrition synergistically. Second, malnourished decompensated patients with cirrhosis are recommended to consume small and frequent snacks and to include a carbohydrate-based late-evening snack in the dietary regimen necessary to spare hepatic glycogen depletion (7, 29–31). However, late food timing has been described to induce decreased energy expenditure, impaired glucose tolerance, and body temperature (32). Mistimed food and sleeps also result in changes in inflammatory markers and plasma proteins in human beings (33). Third, decreased dietary nutrient intake and impaired global protein synthesis have been demonstrated to contribute to sarcopenia in cirrhosis (7). Sarcopenia is a major component of malnutrition. Intriguingly, Nishikawa et al. showed that sleep–wake disturbance is closely associated with sarcopenia especially in cirrhotics (34). Last, it is suggested that cirrhotics have increased daytime levels of melatonin and delayed onset of melatonin peak at night (35, 36). Indeed, disrupted melatonin rhythm might give risk to a biological clock phase-shift, and impaired circadian rhythm might contribute to the pathogenesis of sleep–wake disturbance in cirrhosis (37). Patients with cirrhosis at risk of malnutrition might be deficient in tryptophan, which negatively impacts the biosynthesis of melatonin (38).

Our subgroup analyses indicated that the differences regarding PSQI scores are more significant between low/moderate and high malnutrition risk groups in male patients with cirrhosis, <65 years or with relatively preserved liver function (CTP-A/B or MELD <15). The reasons for these remain elusive, and further studies will be necessary to confirm our results. In view of these results, we provide useful clues and shed light on the targeted population who will be beneficial from the management of sleep difficulties. For instance, zolpidem, a high-affinity positive modulator of ω1 GABAA receptors, might be effective and safe in CTP-A/B patients in improving PSQI score (25).

We acknowledge the following limitations to this study. First, we could not establish causal relationships between sleep–wake disturbance and malnutrition risk. Second, we implemented a self-reported sleep questionnaire rather than objective methods, such as PSG or actigraphy, allowing more precise measurement. However, it should be emphasized that correlations between subjective and objective sleep–wake disturbances are moderate (19). Third, we excluded subjects with severe HE due to lacking reliability in self-reported scale, which might lead to selection bias. Fourth, the sample size was relatively small in the current study. As a matter of fact, the vast majority of previous studies regarding clinical relevance or implication of sleep disorders in patients with advanced chronic liver diseases is based on a small cohort (ranging from 12 to 193 subjects) (4, 27, 39, 40). However, we believe our single-center findings appear to be the first step to instigate a further multi-center investigation. Last, it has been documented that other sleep–wake abnormalities, such as insomnia, excessive daytime sleepiness, and impaired sleep duration, might be correlated with nutritional status. Actually, our research group is now conducting seminal investigations with respect to the clinical implications of these pathologic entities in cirrhotics.

## Conclusion

In conclusion, poor sleep quality is strongly correlated with high malnutrition risk in patients with cirrhosis. Considering sleep–wake disturbance as a remediable complication, it is tempting to incorporate therapies to reverse poor sleep quality aiming at improving nutritional status in cirrhosis.

## Data Availability Statement

The raw data supporting the conclusions of this article will be made available by the authors, without undue reservation.

## Ethics Statement

The studies involving human participants were reviewed and approved by Ethics Committee of Tianjin Medical University General Hospital. The patients/participants provided their written informed consent to participate in this study.

## Author Contributions

YH, XW, ZY, BW, and CS designed the study, analyzed the data, and prepared the original draft. HF, CL, and LM conducted the study and edited the manuscript. XF and LL analyzed the data and reviewed the manuscript. BC, XC, and LS collected the data and conducted statistical analysis. BW and CS designed and monitored the study and made critical revisions to the manuscript. All authors have approved the final draft submitted.

## Conflict of Interest

The authors declare that the research was conducted in the absence of any commercial or financial relationships that could be construed as a potential conflict of interest.

## Publisher's Note

All claims expressed in this article are solely those of the authors and do not necessarily represent those of their affiliated organizations, or those of the publisher, the editors and the reviewers. Any product that may be evaluated in this article, or claim that may be made by its manufacturer, is not guaranteed or endorsed by the publisher.
